# Calendar

**DOI:** 10.1111/iwj.14069

**Published:** 2022-12-28

**NORTH AMERICA**

**26‐30 April 2023**

***Symposium on Advanced Wound Care (SAWC) Spring 2023***

**National Harbor, MD, USA**

www.sawcspring.com

**EUROPE**

**2‐3 February 2023**

***Norwegian Wound Care Society***

**Oslo, Norway**

www.nifs-saar.no/oslo-2023

**April 18, 2023**

***WCS Wound Congress 2023***

**Utrecht, The Netherlands**

www.wcs.nl/congres/wcs-wondcongres-2023

**INTERNATIONAL**

**18‐19 January 2023**

***International Conference on Wound Care ICWC***

**Bangkok, Thailand**

www.waset.org/wound-care-conference-in-january-2023-in-bangkok

**23‐24 February 2023**.

***7th World Congress on Wound Healing and Critical Care***

**Dubai, United Arab Emirates**

www.aast.org

**25‐26 March 2023**

***International Conference on Wound Healing Systems and Technologies (ICWHST*** ***)***

**Tokyo, Japan**

www.waset.org

